# The Research Visit of the Future: an academic-industry model for telehealth, electronic clinical outcomes assessments, and Decentralized Clinical Trials (DCTs)

**DOI:** 10.1186/s13063-025-08886-8

**Published:** 2025-06-06

**Authors:** Noah Goodson, Christopher Wiltrout, Joss Warren, Steven Reis, Mylynda Massart

**Affiliations:** 1 THREAD Research, 2000 Centregreen Way, Suite 300, Cary, NC 27513 USA; 2 University of Pittsburgh, Scaife Hall, Suite 301, 2550 Terrace Street, Pittsburgh, PA 15261 USA

**Keywords:** Decentralized clinical trials, Telehealth, Inclusion, Diversity, Digital divide

## Abstract

The COVID pandemic initially led to the unprecedented suspension of large numbers of clinical research studies and trials that required in-person study visits, highlighting the need to reevaluate how human participant research projects are conducted. During this time the University of Pittsburgh Clinical and Translational Science Institute (Pitt CTSI) embarked on a multi-year initiative to envision the Research Visit of the Future, which ultimately led to an academia–industry partnership that enables the scalable self-service model using a virtual clinical research platform for clinical studies at a major academic research institution. This model enables the flexible deployment of eConsent, Telehealth (audio/video) calls, synchronous and asynchronous data capture, integrated sensors and wearables, and patient engagement tools, all within a single participant-focused research platform. Academic investigators can configure their studies without custom coding across a wide range of study designs, including hybrid- and fully decentralized clinical trials. Here we present the journey to identifying the technology requirements to enrich data collection, structure of our partnership model, and considerations for crossing the digital divide to enable broader access to clinical research among diverse and under-represented communities.

## Introduction

Academic research institutions play a critical role in advancing our understanding of clinical trial methodologies and best practices. Despite their central role in innovation and demonstrating effective trial designs, academic researchers may not always have access to the latest technology available to their counterparts in industry-sponsored studies [1, 2]. We present a model for an academia-industry partnership that allows academic investigators to run technology-enabled studies, inclusive of hybrid and full decentralized clinical trials (DCTs), which have proliferated over the past several years due in large part to the effects of the COVID-19 pandemic. However, the journey to this partnership model did not start with the pandemic or even with knowledge of the terms DCT, remote trial, or virtual trial. Instead, it began with an initiative to envision the “Research Visit of the Future” that focused on how to make research more accessible to patients, community members, and populations.

### Bringing the research visit to the participant

In 2017, the University of Pittsburgh Clinical and Translational Science Institute (Pitt CTSI) began evaluating how to make research more accessible to participants and to address the lack of diversity in clinical research [3–5], which persists today despite years of work to reduce disparities [6–10]. Many individuals lack the opportunity to participate in studies simply because of where they live [11–13]. In western Pennsylvania, where the majority of Pitt CTSI trial participants reside, many are unwilling or unable to navigate barriers to participation such as traveling to and parking at a sprawling urban academic health center campus.

Across healthcare, the use of telehealth (audio/video calls) to reach more remote patients was a growing clinical solution that some institutions were exploring, but concerns about the digital divide and technology availability prevented adoption in research at scale [14, 15]. The Pitt CTSI team began asking if telehealth could be applied to research: Could some studies or parts of studies be conducted utilizing technology, eliminating travel while maintaining data quality and patient safety? Studies had been conducted on a small scale [16, 17], but could these digitally enabled research practices unlock access to research for millions of Americans disadvantaged by the current system?

### Renegotiating risk management

In early 2020, the research world experienced a dramatic upheaval. The COVID-19 global pandemic transformed the research landscape overnight. Nearly every clinical study was forced to put recruitment on hold, alter methodologies, and develop creative mechanisms for collecting data from participants [20, 21]. Heroic efforts were undertaken by many researchers who found ways to continue their studies using novel approaches, such as performing blood draws in parking lots and delivering study medication from the research sites to participants’ homes to ensure continuity of study treatment and participant safety.

The pandemic also forced a re-evaluation of risk by sponsors, sites, and regulatory agencies. The risks associated with adopting technology and the concerns over a digital divide were insignificant compared with the practical reality that participants were either not permitted to visit research sites due to site precautions or were unwilling to risk their own safety by participating in onsite research visits. This change in risk-drivers fast-tracked the adoption of technology from industry, which supported remote technology-based research to get their studies back on track more rapidly than traditionally conducted clinical research [22]. During this time, some advocated that this new remote approach to research would become a best practice standard [19], although recent data suggest a relatively modest change in how trials are leveraging technology post-pandemic [23]. The acceleration in technology awareness and use from the pandemic brought new companies offering previously niche technologies into the mainstream as potential partners in creating the Research Visit of the Future.

### Building an academic-industry model for remote research

Leveraging a diverse group of academics, public health professionals, clinicians, and technology experts, Pitt CTSI began formally assessing the telehealth landscape for technology solutions that could pivot to support remote research more broadly. Pitt CTSI outlined the minimal requirements to ideal features before beginning to search for technology partners to fulfill these needs.

Through this assessment and the insights from investigators, Pitt CTSI came to see the problem as much broader than needing a telehealth platform. During the initial assessment, the team was not formally familiar with the term decentralized clinical trials (DCTs) [18, 19]. When Pitt CTSI launched this initiative, the team was focused on a solution that could support the needs common to clinical trials at an academic research institution (Table 1). These included technology requirements to serve both researchers and participants and included things like electronic consent (eConsent), electronic clinical outcome assessment (eCOA) inclusive of the creation and management of electronic diaries and surveys, electronic source site documents (eSource), data tracking, telehealth (audio/video) visit capacity, and asynchronous data collection. The Pitt CTSI team envisioned a remote workflow in which researchers consent participants, collect data, engage and communicate with participants when and where it was convenient for both investigators and participants. For reasons of practicality and scale, this workflow would need to be relatively quick to deploy and be priced to support academic research budgets. The desired technology would need to also support studies in which some visits are onsite while others are remote. After an extensive vetting process of clinical telehealth platforms, it became evident that modifying these platforms to support research studies was time and cost prohibitive. Therefore, the Pitt CTSI team embarked on a search for partners that created a decentralized platform explicitly designed for clinical research.

**Table 1:** Desired technology solution for researchers and participants to serve the Research Visit of the Future

*Feature*	*Researchers*	*Participants*
*eConsent*	• Deliver consent form(s) to participants in multiple locations • Have option for parental assent—pediatric consent • Review consent forms with patients remotely or in person • Validate signatures and countersign consent forms • Update consent forms during study as needed	• Access and review study and consent materials • Discuss study questions with investigator • Provide informed consent/parental assent • Receive copies of signed consent form(s)
*Data collection*	• Collect data, including patient-reported outcomes (ePRO), clinician-reported outcomes (ClinRO), validated tools/questionnaires, surveys, and diaries • Save instruments and re-use from a library of defined and validated instruments • screen reports to validate with license holders • Ability to collect data from devices and sensors	• Complete surveys or ePRO at home online or via audio–video call with study personnel • Use remote data collection via study devices and sensors • Ability to use sensors or wearables to provide data (e.g., smart watch for heart rate monitoring) • Secure connections to ensure privacy
*Quality control*	• Ability to upload paper documents if required • Full audit trails of data • Query and resolve queries on data • Track adverse event reports and outcomes	
*Engagement*	• Prescheduled and ad hoc reminders and alerts (popup, email, SMS) to single or multiple participants • Track and respond to participant progress and activity • Scheduled/on-demand audio/video calls with participants • Add participants • Change participant status • Review participant activities and status with full audit trails • Push new activities and documents to participants (prescheduled and ad hoc)	• Receive reminders and alerts • Single point of access for all study interactions • Stay up to date on actions required and know when the next activity will be • Receive activities and documents automatically
*User personas*	• Multiple flexible user personas to account for range of study personnel	• Functions for participants, pediatric participants, and caregivers
*Accessible to all users*	• Works on iOS, Android, tablets, phones, and laptop/computer browser • Multiple language options	• Works on iOS, Android, tablets, phones, and laptop/computer browser • Multiple language options • Multiple accommodations (visual, auditory, tactile)
*Study creation*	• Ability to develop and deploy studies without any custom coding • Enable study teams to configure, deploy, and manage studies on their own • Empower teams to manage, monitor, and modify studies on their own • Enable study teams to configure new documents, consents forms, surveys, eCOA, eCRFs, data collection forms, and other eSource documents	

In addressing the needs and ideal capabilities of remote research (Table 1), the CTSI team observed two common models of technology adoption at the University of Pittsburgh that needed to be considered during their evaluation of technology platforms: the Open-Source Model and the Individual-Study Model. These are uniquely reflective of the structures and challenges of managing a large research portfolio within an academic research institution deploying hundreds of studies ranging from massive national registries to small investigator-initiated trials. A third model proposed here is called the Central Hub Model to directly address the institutional challenges in technology deployment within the two existing models.

Study teams use the *Open-Source Model* when their options and budgets are limited. Open-source solutions enable study teams to collect certain types of data, and the low cost of technology procurement and deployment enables teams to focus their budgets on other areas of the research program. Open-source models often leverage institutionally available technology that is effectively free for university researchers. For example, if the study team's organization has a video platform, they may re-appropriate this tool to offer remote visits since the technology is already paid for by the university. This approach lowers the cost of individual research programs but can involve additional effort and risks for managing study data as these tools are not always fit-for-purpose for research.

In an *Individual Study Model*, research teams identify and deploy a technology or a suite of technologies to support the needs of their study. This model provides the advantage of being specific to the study’s needs, but the team must first identify and integrate the appropriate technologies, which may require custom development that could compound costs and timelines. The individual study model is most analogous to industry studies where a sponsor may select vendors on a study-by-study basis to support their research program. In an academic context, this is generally utilized on studies with higher budgets for technology and services. It is primarily differentiated from the open-source model in that teams may use more costly but fit-for-purpose solutions.

After considering these approaches, Pitt CTSI, which provides extensive research infrastructure and services to the health sciences research community at the University of Pittsburgh, envisioned a third *Central Hub Model*. In this solution, Pitt CTSI planned to establish an enterprise partnership with a technology provider and would cover the upfront costs of standing up the technology and transferring knowledge. This would enable the incremental technology cost for individual studies to be low. The challenges in implementing this model were to create a cost-effective per-study pricing structure that supports both academic research budgets and the training and guidance to enable investigator self-service.

There were two requirements for the Central Hub Model partnership to be successful. First, the technology partner would need to support all critical technological needs on a single platform including eConsent, Telehealth, eCOA, eSource data collection, and patient engagement. If technology only supported a subset of needs, multiple partners or tools would be required, undermining the model's effectiveness as an enterprise solution.

Second, and perhaps more important, the technology would need to be rapidly scalable to meet the needs of a research portfolio that includes hundreds of programs at any given point in time. Practically, this would mean that individual studies must not need to be coded by specialists but are capable of being self-configured. Any technology requiring specialized coding or hourly support from the technology partner would become ineffective from a cost perspective for academic research at scale. This solution specifically and directly bridges the digital divide between researchers and the technology needed to engage patients and collect data remotely.

Following an extensive review and vetting process, Pitt CTSI identified a single partner, Thread, who effectively combined both the technology features and the potential for self-service configuration. As the industry continues to evolve, additional companies are deploying similar suites of solutions and developing their own self-service models. Beyond technology, this academia–industry partnership required the additional creation of a specific scalable financial model that would result in low cost to individual researchers while still covering the maintenance and deployment of a cloud-based technology solution (Table 2).

**Table 2:** Common models for deploying technology in clinical trials in an academic research setting

Model	Advantages	Disadvantages	Cost	Researcher labor	Study risks
Open-Sources	Low cost of entry, flexible for many study needs	May not be optimal solution, poor integration, disjointed user experience, can disperse and complicate data, may inject some data integrity and compliance risks that must be actively managed, may not contain all desired features	Low in total financial output	Often very high labor costs for all users including creating the solution, tracking different inputs	Poor experience for participants, poor experience for sites, major burden on all users to manage and navigate various technologies
Individual Study	Study specific needs identified, can work within limited budgets, select best-fit suite of solutions	Can be lots of work to select best solutions, poor integration, often disjointed user experience, can disperse and complicate data	Variable to study, hard to predict	Extensive labor in selecting and managing various service provider	Poor experience for participants, poor experience for sites, major burden on all users to manage and navigate various technologies
Central Hub	Low per study cost, access to top-tier technology, flexible and integrated user-centric solutions	Major institutional buy-in and investment required, knowledge transfer and roadmap to independence can be time-consuming	Greater topline cost for partnership, but low per-study costs, and strong advantage when operating at scale	High upfront labor with staffing commitments required to transfer knowledge with ongoing support, low per-study labor costs that decrease over time with scale and experience	Requires ongoing bi-directional commitment to partnership, academic goals and industry technology goals not always aligned,

### Implementing the research visit of the future

Having formalized our academic–industry collaboration between Pitt CTSI and Thread in the Central Hub Model, we initially deployed five studies (Table 3), with 25 more planned in the next year. As part of this deployment, Pitt CTSI are also collecting data from study teams to understand their expectations, concerns, and wish lists related to the use of a virtual research platform. Pitt CTSI plan to publish user experience and outcomes data in the future as part of our ongoing efforts to establish standards and best practices for the Research Visit of the Future.

**Table 3:** Current studies deployed within the Central Hub Model

Study	Type	Cohort	Visits	Configuration	User personas	Platform tools
1	Observational hybrid DCT	50	10 remote	THREAD-Lead	PI, co-I, CRC	eConsent, 2 ePROs, 7 eCRF tools, prescheduled alerts, telehealth calls
2	RCT hybrid DCT	1000	4 remote, 2 in person	THREAD-Lead	PI, co-I, CRC, DM	eConsent, 3 ePROs, 11 eCRF tools, prescheduled alerts, telehealth calls
3	Observational hybrid DCT	250,000	1 in person	THREAD-supported	PI, co-I, CRC	eConsent
4	RCT DCT	600	3 remote	Self Service	PI, co-I, CRC	eConsent, 4 ePROs, 7 eCRF tools, prescheduled alerts, telehealth calls
5	RCT DCT	30	6 remote	Self Service	PI, co-I, CRC	eConsent, 13 ePROs, 2 eCRF tools, prescheduled alerts, telehealth calls

Deployed studies have included features such as eConsent, Telehealth, surveys, validated eCOA instruments including patient and clinician reported outcomes, and engagement tools like patient reminders and alerts. An unexpected aspect of the partnership was the demand for electronic clinical outcomes assessments (eCOA) from Pitt CTSI researchers. Within the partnership, a collaborative pivot was made to facilitate this demand from researchers by providing additional training and support for the Pitt CTSI team to be able to configure and license instruments to specification. Because eCOA instruments may have specific validation and deployment requirements beyond those of a simple survey, and eDiary training from Thread and additional clear lines of accountability were established.

The first deployed studies were led by the technology partner, with the Pitt CTSI team engaging to learn the process. The next study launched was a supported study where the Pitt CTSI team configured the study, and the technology partner provided feedback and insights to ensure best-practices were followed. Finally, Pitt CTSI has been able to configure the first two Self-Service studies.

*RCT*, randomized clinical trial; *DCT*, decentralized clinical trial; *PI*, principal investigator; *co-I*, co-investigator; *CRC*, clinical research coordinator; *eRPO*, electronic patient-reported outcomes; *eCRFs*, electronic case report form.

#### Facing a new digital divide

The Pitt CTSI team set out with the objective of creating more inclusive research with the hypothesis that technology could help achieve the goal of creating the Research Visit of the Future. In this effort, the digital divide was the most commonly cited objection to utilizing technology solutions throughout the clinical research process [24, 25]. The argument is that disadvantaged participants, particularly those in rural or resource-poor communities, will not have access to web-enabled devices, internet service, or technology literacy, and this will prevent them from participating in research studies where technology is necessary. This is true in some circumstances [26], but Pitt CTSI’s detailed investigation into the technology options available and researcher hands-on experience suggests this is not always the case, with 95% of Americans now having regular broadband internet access and the “rural” population only lagging behind the urban by 2% (93% broadband access at home) [27–29]. It remains true that marginalized communities, including certain racial or ethnic identities and those with lower education or income, have lower smartphone ownership rates, but today 90% of Americans own a smartphone, and that number continues to grow across all groups [30].

A decade ago, the gap in technology access for historically disadvantaged populations was much larger—today it has narrowed, with meta-analysis showing rural patients are highly satisfied with telehealth [31, 32]. When dissatisfaction is reported, it is typically related to either relationship building with a clinician or, most commonly, frustrations with the maturity of technology itself [33]. It remains common to suggest the digital divide is a barrier to technology-enabled trials and DCTs. There is still a digital divide for some participants, but evidence suggests it has decreased. Furthermore, many participants impacted by this divide are even more strongly impacted by unequal access to medical care and research opportunities. Technology may, in fact, be the only way some participants can join in research. We have hypothesized that the prevalence of internet and smartphone access and the published objections of patients suggests that, in many cases, the digital divide has shifted.

In the view of the authors, there is another digital divide in clinical trials. It is not simply the lack of participant internet or technology access and fluency, though in some specific cases this remains true. Rather, this hidden digital divide is the fractured deployment systems and technologies available to sponsors, investigators, and research sites. We, the researchers, are the ones with a digital divide: In the traditional clinical research model, it is the participants who bear the burden of transportation across geographic distance, time lost in waiting rooms, and disconnection from busy research sites. In this hidden digital divide, it remains the participants who experience the significant personal cost and barriers to research which persist when technology is not effectively integrated into the way research is conducted.

This digital divide of researchers and technology creates significant resistance to the adoption of new methodologies, though rarely from participants. Collecting surveys on paper may be inferior from a data integrity and participant experience perspective, but it may well appear simpler and more affordable to a research investigator or sponsor. The adoption of any new technology, and particularly a suite of technologies, involves risk tolerance and the creation of new standard operating procedures to account for the new best practices. When disjointed technologies are combined, there may also be a compounding effect of research site/staff burden in technology adoption [34]. The data presented here are the first steps to developing best-practices within the newly established Central Hub Model to cross this digital divide and reach research participants.

## Conclusion

Creating greater equity in research starts with access. Geographic and socio-economic barriers will take time to erode. No collaboration or technology will solve these challenges alone, but when used properly, the right collaborative deployment of technology may enable a greater number of participants to engage in research. We have much to learn about how specific technology solutions to enable remote research, such as electronic surveys or telehealth calls, impact the participation of specific populations and the efficiency with which clinical research is conducted. As part of our pilot trial of the Central Hub Model, the team will learn whether and why investigators prefer digital versus paper and remote versus in-person data collection in understanding whether a real or perceived digital divide prevents investigator acceptance and deployment of hybrid and full DCT approaches to clinical research. Pitt CTSI will also monitor participant recruitment, enrollment, engagement, and retention to evaluate how and for whom our envisioned Research Visit of the Future works best and why. Indeed, results may reveal new aspects to the digital divide that were previously under-researched.

Our hope is that this academic–industry partnership provides a pathway to identify and refine best practices in technology deployment within research. If it is the research sponsors and sites who are facing a digital divide, then it is our responsibility to build bridges to cross over to patients, enabling access to research for everyone, everywhere.
